# Paraphrenia - forgotten or undiagnosable psychosis?

**DOI:** 10.1192/j.eurpsy.2023.2231

**Published:** 2023-07-19

**Authors:** E. Lukacs, A. Nireștean, A. Sima-Comaniciu, L. M. Muntean

**Affiliations:** 1 Psychiatry, George Emil Palade University of Medicine, Pharmacy, Science, and Technology of Targu Mures, 540136 Targu Mures, Romania, Târgu Mureș, Romania

## Abstract

**Introduction:**

Paraphrenia is a chronic psychosis that has generally lost its status as an independent nosological entity, not being included in DSM-5. From the perspective of the particular psychopathological picture and the impact of the disease on the patient’s functioning in roles, paraphrenia remains a challenge for the clinician in terms of nosological classification and the correct therapeutic approach.

**Objectives:**

We take into account a patient who presents the classic diagnostic criteria of paraphrenia with a clinical and evolutionary picture followed for 15 years, with the aim of bringing the paraphrenic phenomenology back to the fore.

**Methods:**

The case presentation will focus on the richness and absurdity of the delusional ideas that are in great contrast with the good insertion into reality of the subject and the preservation of the core of the personality. We will also describe the main landmarks of positive and differential diagnosis.

**Results:**

We believe that paraphrenia deserves to be differentiated from other psychotic disorders through the particular variant of insight that also explains the significant capacities of dissimulation and as a result of insertion into the roles of life. This attribute also explains the increased potential for danger and unpredictability of the paraphrenic patient.

**Conclusions:**

Our approach is an argument for the psychopathological understanding of paraphrenic psychotic phenomenology independent of different nosographic classification systems. We are trying to contribute to increasing the quality of differential diagnosis in psychoses.

**Keywords:**

psychosis, paraphrenia, differential diagnosis

**Disclosure of Interest:**

None Declared

